# Bmk-1 regulates lifespan in *Caenorhabditis elegans* by activating hsp-16

**DOI:** 10.18632/oncotarget.4618

**Published:** 2015-07-31

**Authors:** Hong Qian, Xiangru Xu, Laura E. Niklason

**Affiliations:** ^1^ Department of Anesthesiology, Yale University School of Medicine, New Haven, CT 06520, USA; ^2^ Max Planck Institute for Biology of Ageing, 50931, Cologne, Germany

**Keywords:** Geotarget, BMK-1, longevity, aging, stress response, hsp16

## Abstract

The genetics of aging is typically concerned with lifespan determination that is associated with alterations in expression levels or mutations of particular genes. Previous reports in *C. elegans* have shown that the *bmk-1* gene has important functions in chromosome segregation, and this has been confirmed with its mammalian homolog, KIF11. However, this gene has never been implicated in aging or lifespan regulation. Here we show that the *bmk-1* gene is an important lifespan regulator in worms. We show that reducing *bmk-1* expression using RNAi shortens worm lifespan by 32%, while over-expression of *bmk-1* extends worm lifespan by 25%, and enhances heat-shock stress resistance. Moreover, *bmk-1* over-expression increases the level of *hsp-16* and decreases *ced-3* in *C. elegans*. Genetic epistasis analysis reveals that *hsp-16* is essential for the lifespan extension by *bmk-1.* These findings suggest that bmk-1 may act through enhanced hsp-16 function to protect cells from stress and inhibit the apoptosis pathway, thereby conferring worm longevity. Though it remains unclear whether this is a distinct function from chromosomal segregation, bmk-1 is a potential new target for extension of lifespan and enhancement of healthspan.

## INTRODUCTION

The BimC subfamily of kinesin-1 (bmk-1) gene belongs to a large super family of motor proteins that participate in various critical biological processes, including mitosis and intracellular transport of vesicles and organelles [1]. Kinesin consists of a long coiled-coil stalk with a cargo binding tail at one end, and a globular tail domain at the other end. The highly conserved motor domain, 320 residues in size, contains both microtubule and nucleotide binding sites. Kinesin proteins are localized to centrosomes, spindle microtubules, and the spindle midzone, and act during the early stages of mitosis to facilitate centrosome separation and bipolar spindle assembly. These processes are essential for accurate chromosome segregation and progress through the cell cycle [2]. In *C. elegans*, bmk-1 has also been reported to serve a novel function as a “brake” that slows down the rate of anaphase spindle-pole separation [3–5]. The bmk-1 homolog in *Drosophila* contributes to a range of functions in mitosis, all of which are consistent with it exerting outward forces on spindle poles by sliding microtubules relative to a static spindle matrix, or by crosslinking and sliding apart adjacent pairs of antiparallel interpolar microtubules [6].

KIF11, the mouse and human equivalent of the *C. elegans* bmk-1, is one out of 14 kinesin subfamilies which are classified by the phylogenetic analysis for the motor domain. They normally play two major functions in eukaryotic cells, since they participate in all stages of cell division, as well as in intracellular vesical and organelle transport [7–10]. KIF11 normally functions as a mitotic cell cycle and checkpoint regulator [11]. Timely and accurate assembly of the mitotic spindle is critical for the faithful segregation of chromosomes, and centrosome separation is a key step in this process. Premature separation of centrosomes decreases the requirement for KIF11 in spindle assembly, accelerates mitosis, and decreases the rate of chromosome mis-segregation [12]. Tao and colleagues reported that induction of apoptosis in cells treated with a kinesin protein inhibitor (KSP-I) occurs after long-term mitotic arrest [13]. In their studies, KSP-IA, a dihydropyrrole small molecule, arrests cells in mitosis and induces apoptosis by a caspase-dependent pathway [14]. Moreover, KSP-IA was able to induce apoptotic cell death in a p53-independent manner, suggesting that KSP inhibitors could be active in p53-deficient tumors [15]. However, neither bmk-1 nor KIF11 has ever been linked to aging or to lifespan in either mammals or lower organisms. In studies where we modulated expression of *bmk-1* in *C. elegans*, we found evidence indicating that bmk-1 may play an important role in lifespan determination, and that bmk-1 may be acting via hsp-16 for this effect.

## RESULTS

### *Bmk-1/KIF11* is an evolutionarily conserved gene, and the expression of *bmk-1/KIF11* declines with mammalian tissue aging

**Figure 1:**
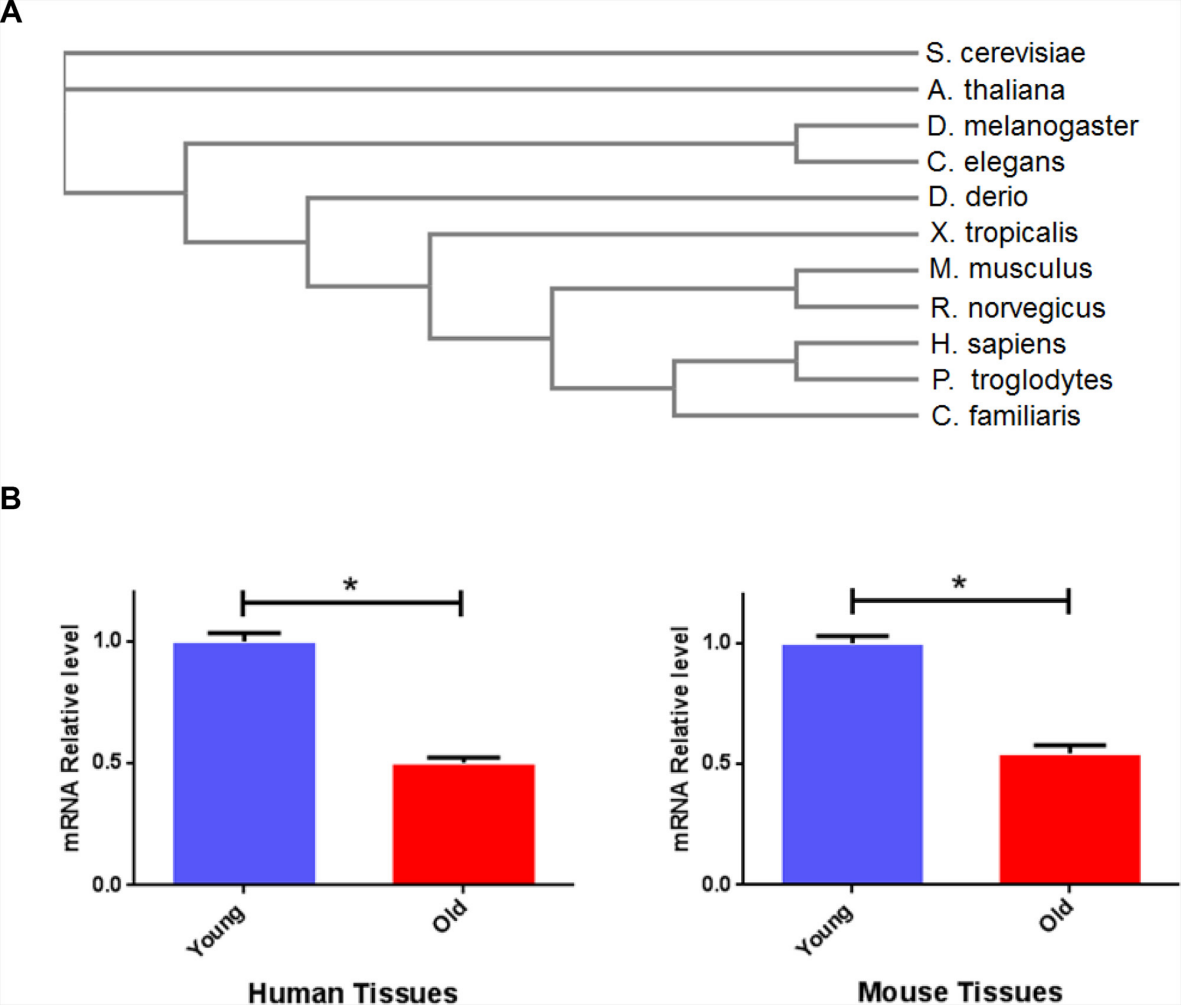
Bmk-1 is an evolutionarily conserved gene and its expression declines with tissue aging across species **A.** Sequences alignment of bmk-1 protein across species ranging from yeast to human. **B.** KIF11 mRNA expression changes with age in human and mouse. Y-axis represents the relative expression level of KIF11 was normalized to ß-actin, and *n* > 3 for each age group. * indicates *p* < 0.05.

To understand the evolutionary conservation of the *bmk-1 gene*, we examined the homology of bmk-1 protein sequences among various species using the NCBI RefSeq database (http://www.ncbi.nlm.nih.gov/refseq/). We then performed a phylogenetic analysis using EMBL-EBI Clustal Omega [16] to assess the protein sequence alignment of bmk-1 and its homologs. Bmk-1, similar to pch-2 [17], is an evolutionarily conserved gene with a Myosin and Kinesin motor domain, which belongs to the P-loop_NTPase family and is found across species including yeast (*S. cerevisiae*), worm (*C. elegans*), fly (*D. melanogaster*), zebrafish (*D. rerio*), rodent (*R. novegicus*, *M. musculus*) and human (*H. sapiens*) (Figure 1a & Supplementary Table S1). As a mitotic cell cycle and checkpoint regulator, bmk-1 is critical for the faithful segregation of chromosomes. This requires an evolutionarily conserved function for *bmk-1* in both recombination and in the formation of more complex chromosome structures [18]. Hence *bmk-1* provides a basic function across the animal kingdom.

To determine the pattern of expression of bmk-1/KIF11 across species and as a function of age, we examined gonadal and brain tissues from mouse and human samples. We measured expression levels of *KIF11* mRNA by affymetrix array analysis in murine and human specimens as described previously^10^. We quantified gene expression in young subjects: 3-month old mice (*N* = 5) from two aging colonies, C57B/6 and DBA2, as well as in 18–25 year-old humans (male and female, *N* = 8). We also examined aged subjects: 22-month old for mice from two aging colonies (*N* = 5 for each colony), and humans >60 years old (*N* = 10). We found that the expression of *KIF11* declines significantly with physiological aging in gonadal and brain tissues of both human and mouse (Figure 1B). This was judged by both fold change in mRNA expression, as well as by *p* values (*p* < 0.01). This observation suggests an evolutionarily conserved loss of expression of this *bmk-1* homolog with tissue aging across mammalian species. Based upon these observations, we asked whether *KIF11* plays a role in longevity. Given that lifespan in *C. elegans* is only 3–4 weeks, as opposed to approximately 2–3 years in mice [19–20], we elected to examine the impact of the *bmk-1* gene expression on lifespan in *C. elegans*.

### Expression of *bmk-1* regulates lifespan of *C. elegans*

**Figure 2:**
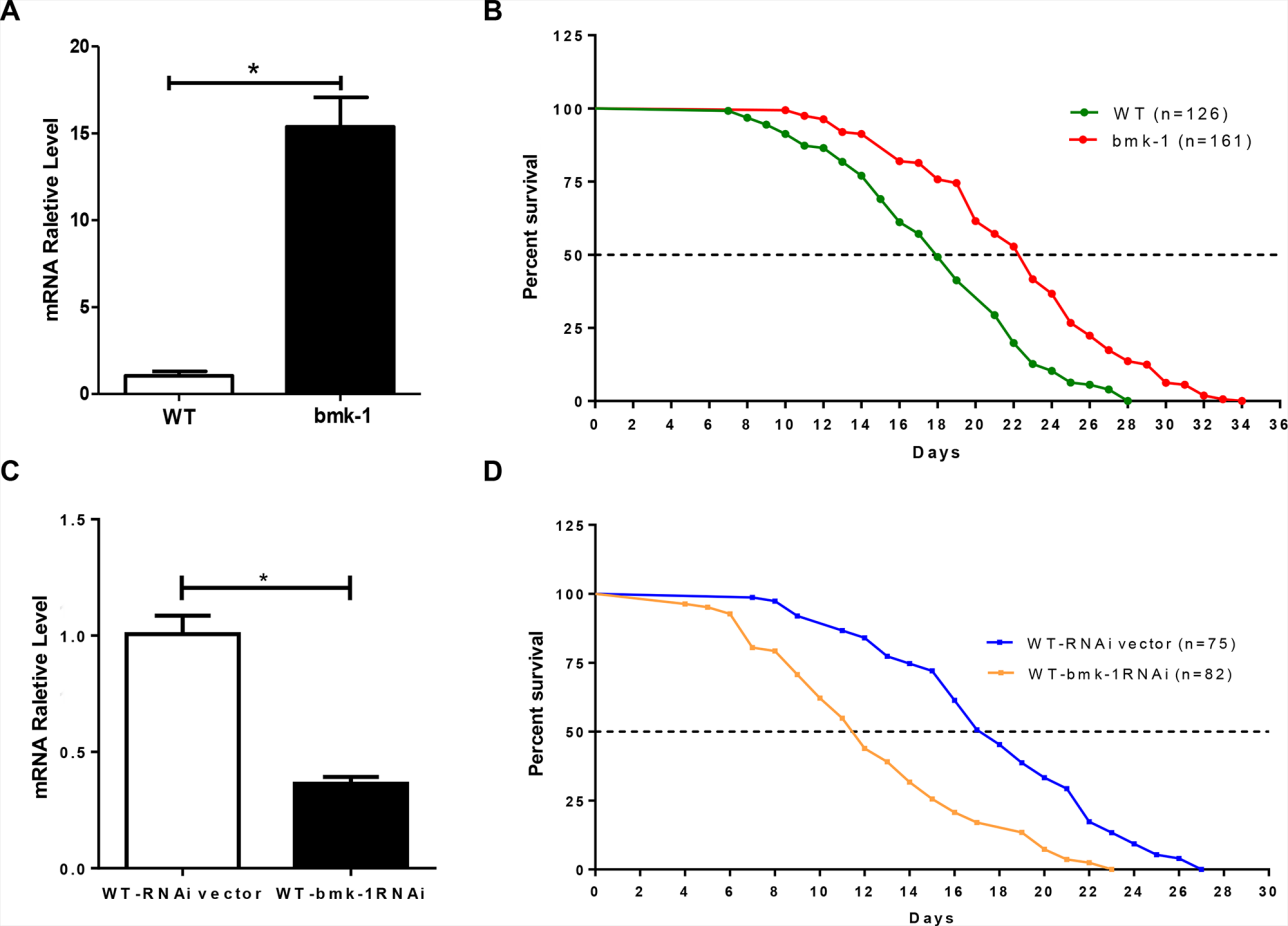
*Bmk-1* over-expression extends lifespan and *bmk-1* inhibition by RNAi shortens lifespan in *C. elegans* **A.** qRT-PCR validation of *bmk-1* over-expression worm lines shows increased *bmk-1* gene expression (* indicates *p* < 0.01). Y-axis represents the relative expression level of bmk-1 normalized to act-1, and *n* ≥ 10 for each group. **B.** lifespan measurement for both WT (GFP-expressing, *n* = 126) and *bmk-1* over-expressing (*n* = 161) animals. Both median lifespan and maximum lifespan of *bmk-1* over-expressing lines show a 25% extension when compared to WT lines (*p* < 0.001, *p* values were derived from student *t*-test and log-rank test). **C.** qRT-PCR validated the RNAi effect, indicating a significant reduction (∼64%) of *bmk-1* transcripts in WT worms treated with *bmk-1* RNAi. Y-axis represents the relative expression level of bmk-1 normalized to act-1, and *n* ≥ 10 for each group. * indicates *p* < 0.01. **D.** A shortened lifespan, both median (32%) and maximum (15%), was induced by *bmk-1*-specific RNAi in *C. elegans* (*n* = 82), as compared to RNAi vector lines (*n* = 75) (*p* < 0.0001).

We first generated *bmk-1* over-expressing *C. elegans* lines that co-expressed green fluorescent protein (GFP) by a microinjection method (Supplementary Figure S1), and studied the functional impact of *bmk-1* on worm longevity. To identify over-expressing *bmk-1* lines, we selected those animals co-expressing GFP by direct visualization. In GFP-expressing lines, we also measured the expression levels of *bmk-1* using quantitative reverse transcription polymerase reaction (qRT-PCR). We distinguished between the expression of endogenous and exogenous *bmk-1* by using an expression vector-specific primer and a *bmk-1*-specific primer (Supplementary Figure S2). In GFP-positive over-expressing lines, the level of exogenous *bmk-1* expression was ten times higher than the level of endogenous *bmk-1* in the wild-type (WT, GFP-expressing) controls (Figure 2A). After confirming over-expression of *bmk-1* in worm lines, we then subjected *Bmk-1* over-expressing worms and WT worms to lifespan measurement ^13^. The median lifespans of WT and *bmk-1* over-expressers were 18 days and 22 days, respectively, with maximum lifespans of 28 days and 34 days, respectively (Figure 2B). Hence, *bmk-1* over-expressing lines extended both median and maximum lifespan by approximately 25% as compared to WT (log rank test, *N* = 126/161, *p* < 0.001).

We next examined lifespan after RNAi-induced *bmk-1* knockdown in WT (with GFP) animals. A *bmk-1* specific RNAi clone F10B5.5 was used to feed WT worms, thereby reducing *bmk-1* expression. Only 36% expression levels of *bmk-1* remained after *bmk-1*-RNAi treatment, as assessed by qRT-PCR (Figure 2C, *p* < 0.01). The median lifespan of WT animals was 17 days, but this was shortened to 11.5 days after *bmk-1* RNAi treatment. Maximum lifespan shortened to 23 days from 27 days, and both median and maximum lifespan were significantly shortened by *bmk-1* knockdown (*p* < 0.0001) (Figure 2D). Similar results were also observed from RNAi inhibition in *bmk-1* over-expressing worm lines (Supplementary Figure S3).

### Bmk-1 over-expressing lines have enhanced stress response

**Figure 3:**
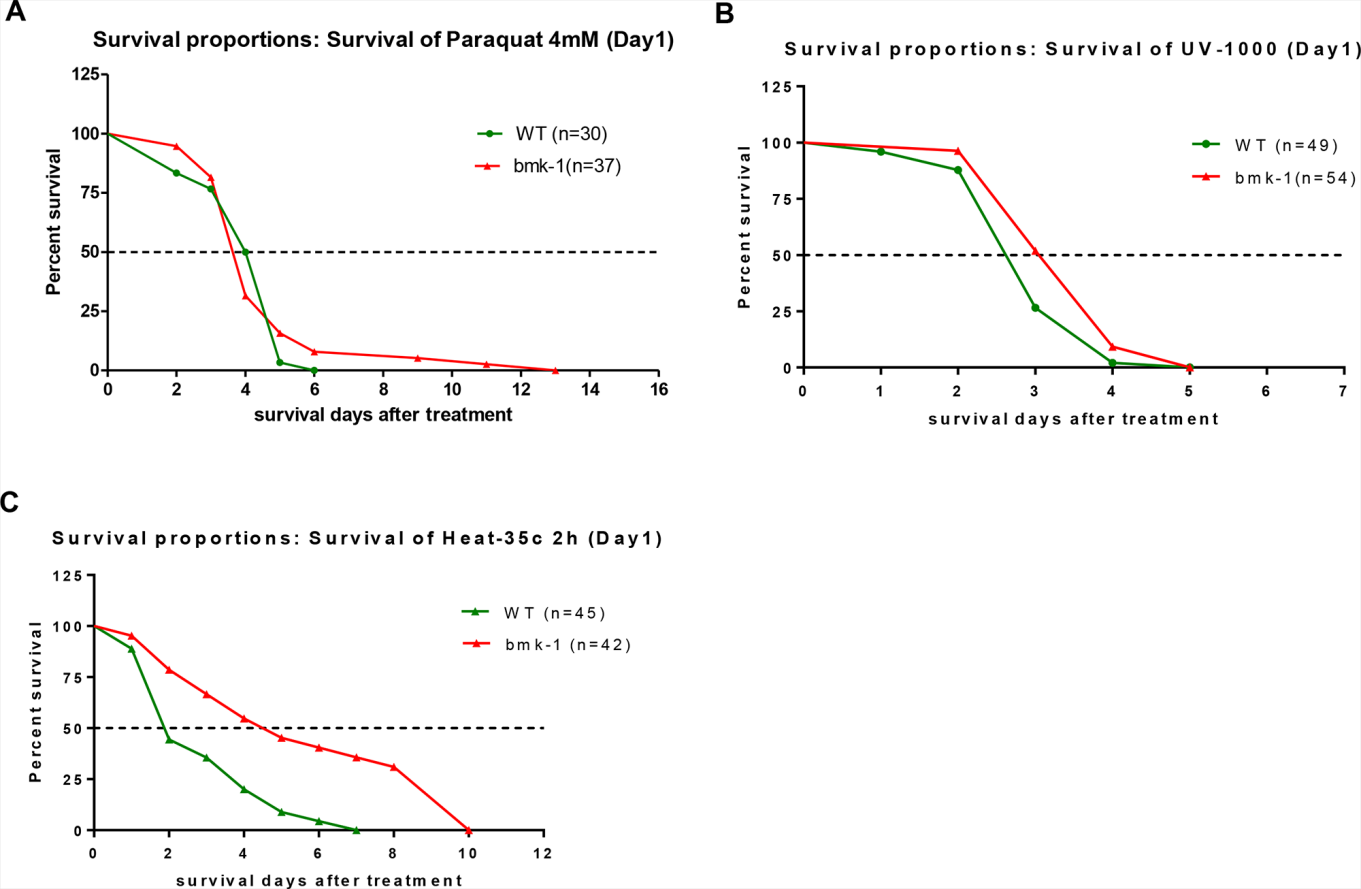
Bmk-1 over-expression lines enhances the stress response in *C. elegans* **A.** After 4 mM paraquat treatment, the median survival of *bmk-1* over-expressing lines (*n* = 37) was not changed as compared to WT (GFP-expressing) lines, but the maximum survival of *bmk-1* over-expressing lines was increased 115% (*n* = 30) (*p* < 0.001). **B.** The median survival of *bmk-1* over-expressing lines (*n* = 54) was increased 15% after UV radiation when compared to WT (*n* = 49) (*p* < 0.01). **C.** With heat shock, the median and maximum survivals of *bmk-1* over-expressing lines (*n* = 42) were increased by 110% and 43%, respectively, when compared to WT (*n* = 45) (*p* < 0.001).

We then determined whether *bmk-1* over-expressing lines could withstand various stressors better than WT (GFP-expressing) controls. Young adult worms that were *bmk-1* over-expressors and WT controls were treated with the oxidative stressor paraquat at 4 mM for their lifespan duration. The median lifespan of *bmk-1* over-expressing lines and the control lines were all about 4.0 days. However, the maximum lifespan of *bmk-1* over-expression and WT lines were 13 days and 6 days, respectively, which is an increase of 120% in maximal lifespan for *bmk-1* over-expressers (*p* < 0.001 maximal lifespans as compared to WT controls by log rank test) (Figure 3A).

To provide a DNA damage stressor, worms were exposed on day 1 to UV radiation at 0.1 J/cm^2^. The median lifespan of *bmk-1* over-expressing lines was 3.0 days and that of control lines was 2.6 days after UV radiation (20% higher for *bmk-1* over-expressers, *p* < 0.01), while the maximum lifespans were 5 days for both (Figure 3B). Lastly, heat shock was administered at 35°C for 2 hours, after which animals were removed to routine conditions and their lifespans tallied. Intriguingly, the median lifespan of *bmk-1* over-expression lines was 130.0% longer than that of control lines (4.4 days vs. 1.9 days) (*p* < 0.0001), and the maximum lifespan of *Bmk-1* over-expression lines was increased by 43% as compared to that of control lines (10 days vs. 7 days) (Figure 3C). Hence, *bmk-1* conferred some lifespan extension in the setting of multiple stressors which affected both DNA and protein integrity [21], but the response to heat-shock was greater than that observed for the other two stressors, and was highly significant. These results imply that *bmk-1* enhances stress-coping capacities of worm, particularly with regard to heat-shock, and thereby extends the lifespan.

### *Hsp-16* is involved in the longevity function of *bmk-1* in *C. elegans*

**Figure 4:**
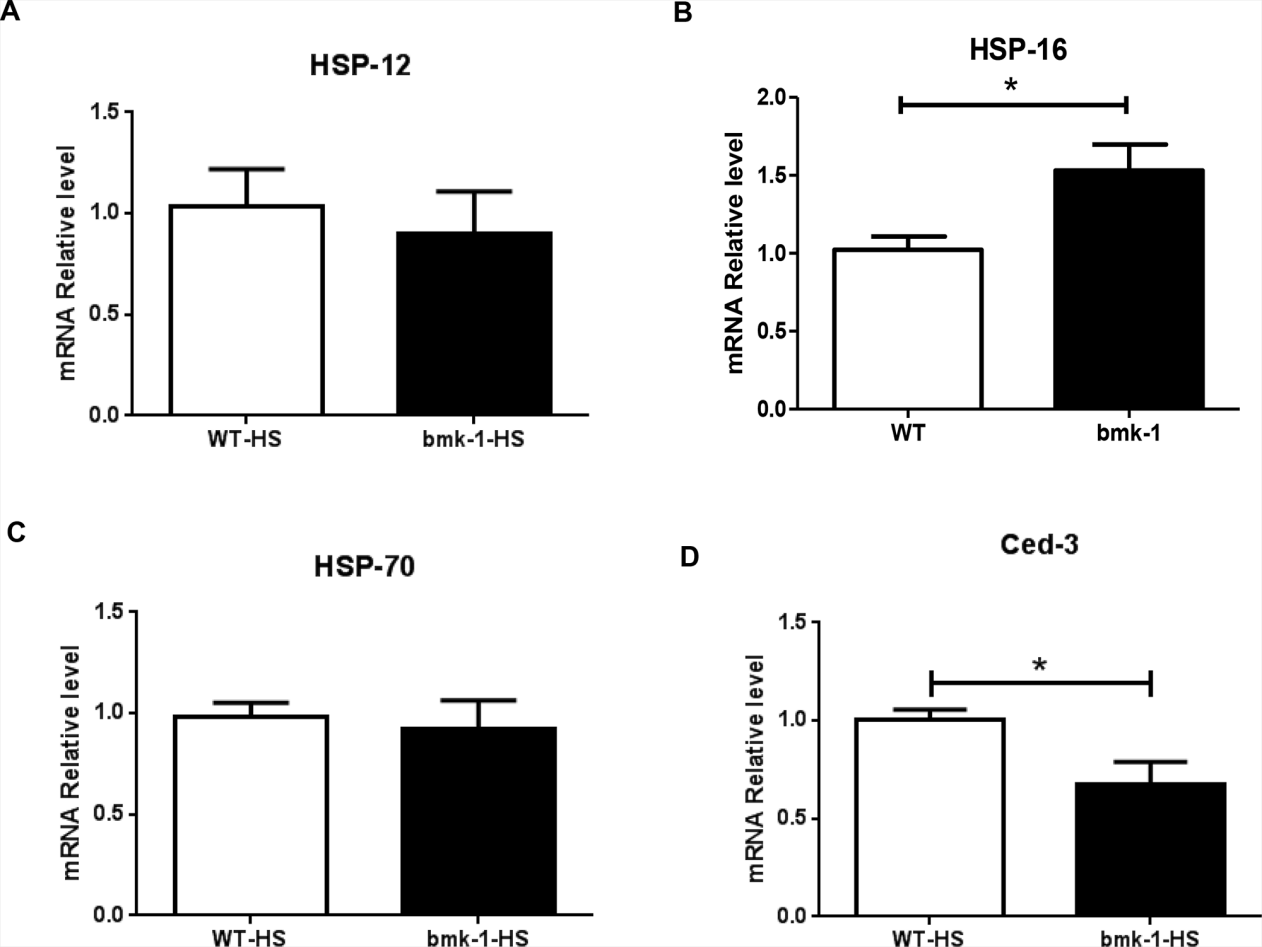
*Hsp-16* expression was elevated after heat shock in *bmk-1* over-expressing *C. elegans* **A.** Expression of *hsp-12* was not changed significantly between WT and *bmk-1* over-expressing lines. **B.** Expression of *hsp-16* increased significantly in *bmk-1* over-expression lines when compared to WT control lines (*p* < 0.05). **C.** Expression of *hsp-70* was not changed much between WT and *bmk-1* over-expression lines. **D.** Expression of *ced-3* decreased significantly *bmk-1* over-expression lines when compared to WT control lines (* indicates *p* < 0.05). Y-axis represents the relative expression levels of hsp12, hsp16, hsp70, and ced-3 normalized to act-1, respectively; *n* ≥ 10 for each group.

Since *bmk-1* over-expression seemed to confer the greatest resistance to heat shock as compared to other stressors we tested, we speculated that key components of heat-shock proteins and/or apoptosis pathways may be involved in this beneficial survival effect. Therefore, we measured the expression of several well-known heat shock proteins (HSPs), as well as the cell death molecule in *C. elegans - ced-3*, the core apoptotic cell death executioner, after heat-shock. [22]. Among three measured HSPs, *hsp-12, hsp-16 and hsp-70*, we found that *hsp-16* expression was significantly increased in *bmk-1* over-expressing lines as compared to control lines, while the expression levels of *hsp-12* and *hsp-70* did not differ significantly (Figure 4). Furthermore, expression of *ced-3* was reduced significantly in *bmk-1* over-expressing lines under baseline conditions as compared to WT controls (Figure 4A–4D). Since over-expression of *hsp-16* has been reported to extend lifespan in worms [23–24], and activation of caspases such as *ced-3* promotes cell death [25], our results suggest that the hsp-16 pathway is affected by *bmk-1* and may contribute to the lifespan extension observed in these studies.

**Figure 5:**
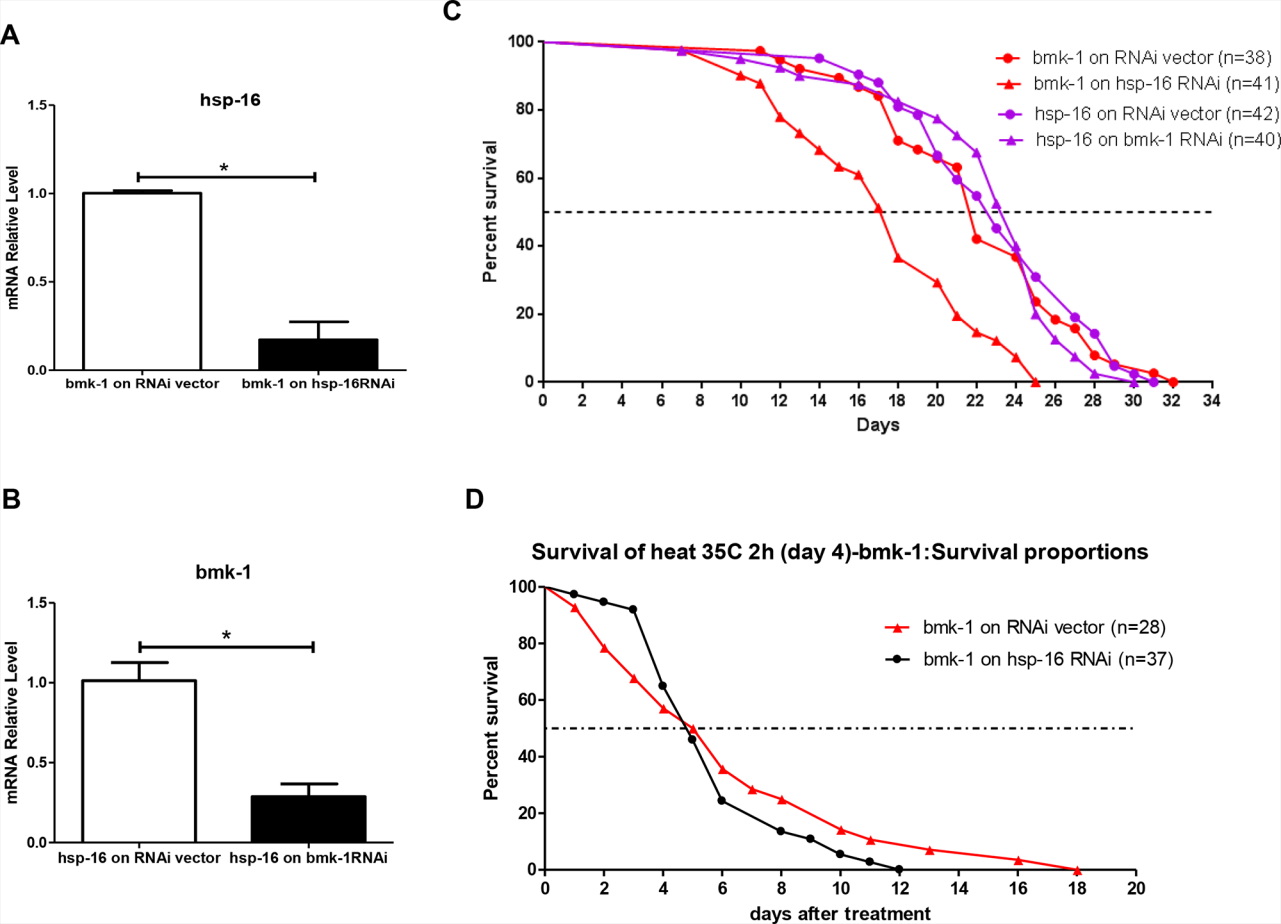
RNAi inhibition of *hsp-16* in *bmk-1* over-expressing worms shortens lifespan and weakens heat shock response **A.** RNAi for *hsp-16* in *bmk-1* over-expression worms resulted in significantly reduced expression by over 80% as validated by qRT-PCR. Y axis represents the relative expression level of *hsp-16* as it was normalized to act-1, and *n* ≥ 10 for each group (*p < 0.001). **B.** Similarly, RNAi for *bmk-1* in *hsp-16* over-expressing worms resulted in significantly reduced expression by over 70% as validated by qRT-PCR. Y axis represents the relative expression level of *bmk-1* as it was normalized to act-1, and *n* ≥ 10 for each group (*p < 0.001). **C.** Red curves show lifespans of *bmk-1* over-expression worms, while purple curves show lifespans of *hsp-16* over-ex. pressing worms. In *bmk-1* over-expressing worms, *hsp-16* RNAi significantly decreased lifespan (77% and 78% decrease in median and maximum lifespan, respectively) (*p* < 0.0001 for both). However, in *hsp-16* over-expressing worms, *bmk-1* RNAi did not change either median or maximum lifespans. **D.** In *bmk-1* over-expressing worms, *hsp-16* RNAi significantly deteriorated worms response to heat shock with a shortening of 1/3 maximum lifespan (*p* < 0.0001, *p* values were derived from student *t*-test and log-rank test. *n* = 28 for RNAi vector group and *n* = 37 *for hsp-16* RNAi group).

To further investigate whether *hsp-16* is a key mediator in *bmk-1* effects on lifespan, we applied genetic epistasis studies with specific RNAis on *hsp-16* in *bmk-1* over-expressing worm lines and on *bmk-1* in *hsp-16* over-expressing lines. We first validated that the effectiveness of *hsp-16* RNAi for in *bmk-1* over-expressing worms. There was a significantly reduced expression of *hsp-16* by over 80%, in *bmk-1* overexpressors that were exposed to hsp-16 RNAi (Figure 5A). RNAi treatment for *bmk-1* in *hsp-16* over-expressing worms resulted in a decrease of 70% of expression levels for *bmk-1*. (Figure 5B). The genetic epistasis investigations showed that reducing *hsp-16* expression in *bmk-1* over-expressing worms significantly decreased both median and maximum lifespans of these animals (77% and 78%, respectively) (*p* < 0.0001). However, reducing *bmk-1* expression by RNAi in *hsp-16* longevity worms did not change either median or maximum lifespans of *hsp-16* worms (Figure 5C). These results suggest that hsp-16 is essential for bmk-1's lifespan regulation in worms, and may work downstream of bmk-1. In contrast, in animals that already constitutively over-express hsp-16, the *bmk-1* knockdown does not affect their lifespan (Figure 5C).

Lastly, we tested whether knocking-down *hsp-16* by RNAi in *bmk-1* over-expression lines would affect the stress coping capacity of this worm line. Heat shock was administered at 35°C for 2 hours, after which animals were removed to routine conditions and their lifespans tallied. The maximum survival of *hsp-16* RNAi treated *bmk-1* over-expression lines was 33% shorter than that of control lines (12 days vs. 18 days) (*p* < 0.0001) (Figure 5D). This indicates that the heat-shock resistance capacity that is acquired by *bmk-1* over-expressing worms acquired heat-shock resistance capacity is indeed relying on the expression level of *hsp-16*. Taking together, hsp-16 is suggested the key mediator for bmk-1′s lifespan extension function by conferring a significant enhancement of stress-coping ability.

## DISCUSSION

Genes that are essential for development and growth are highly conserved in evolution, and the evolutionary conserved genetic pathways such as Insulin/IGF-1 and TOR have been reported to determine normal lifespan in animals ranging from yeast, worm, and fly to rodents [26]. This suggests that both development and aging are essential for survival and evolution in animal kingdom. It is reasonable that some genes may play roles in both development process and aging or lifespan determination. Indeed, there are a few identified genes that are not only crucial for development, but also associated with lifespan determination. For instance, both *daf-2* and *daf-12* affect the dauer larva developmental formation, as well as adult lifespan, in an allele-specific manner in worms [27–28]. In early *C. elegans* embryonic stages, bmk-1 localizes to the region of overlapping interpolar microtubules and functions as a regulator that governs the rate of spindle elongation, as well as the chromosome segregation [4–6]. Our study is the first to suggest that bmk-1 also regulates the lifespan in worms, and that the expression of KIF11, the mammalian homolog of bmk-1, declines with mouse and human tissue aging.

The mechanism by which bmk-1 extends lifespan may possibly be through enhanced expression of *hsp-16* and inhibition of *ced-3* as suggested by the genetic epistasis study. It appears that *hsp-16* is essential for *bmk-1*-induced the lifespan extension, and also accounts for the enhanced heat shock stress resistance of *bmk-1* worms. Heat shock proteins, also called molecular chaperones, are well-known mediators of stress resistance [29]. A large body of evidence accumulated in senescence models at the cellular and whole animal level has consistently shown that heat shock gene expression, in response to various stressors, is poorly induced in aged subjects [30–31]. *Hsp-16* is one of the small chaperone proteins and has been observed to have elevated expression in long-lived worms such as the *daf-2* and *age-1* mutants [32–34]. In addition, transgenic overexpression of *hsp-16* in *C. elegans* increases worm life span [23–24]. These observations have already implicated a link between hsp-16 and lifespan regulation by protecting cells from stress. *Ced-3*, the equivalent of mouse and human caspase-1, is a key regulator that promotes cell apoptosis [22, 25]. The reduction of ced-3 expression in *bmk-1* over-expressing worms suggested an inhibition of the cell apoptosis program, which may stem from increased cellular protection in the setting of increased *hsp-16*, and which may also contribute to a longer lifespan.

In brief, we have described bmk-1 as having a potential new role in lifespan extension in *C. elegans.* This gene effectively extends lifespan in over-expressing lines under a variety of stressors, and knockdown of the gene results in shortening of lifespan. The mechanism of action of bmk-1 is likely mediated by enhancing the expression *hsp-16*. The longevity effects of *bmk-1* and its homologs should be studied in other mammalian systems with cutting-edge genetic approaches.

## MATERIALS and METHODS

### Nematode strains and maintenance

The *C. elegans nematode* strain N2 used for all the experiments was a gift from the Reinke lab. All the *C. elegans* stocks, including constructed strains, were maintained at 25°C on nematode growth medium agar (NGM) plates seeded with *E. coli* strain OP50, as described in our previous study [17, 35]

#### Molecular cloning

Multiple DNA fragments were cloned by using Gateway three-fragment vector construction kit. (Invitrogen, Carlsbad, CA).

**Step 1**: Produce three fragments with flanking site by PCR:

Three fragments: promoter Myo-3 (gift from Koelle lab), gene GFP or *bmk-1*, and 3′-UTR were amplified by PCR using primers that incorporate flanking attB4 and attB1r sites in fragment myo-3, flanking attB1 and attB2 sites in the GFP or *bmk-1* genes, and flanking attB2r and attB3 sites in 3′-UTR. Templates for amplifying Myo-3, GFP, and 3′-UTR are others’ plasmids (gift from Reinke lab), and the gene *bmk-1* was amplified from *C. elegans* genomic DNA (gift from Reinke lab).

**Step 2**: Entry clones were generated by BP reaction:

Three PCR products from step 1 and three donor vectors P4-P1r, P1-P2, P2-P3 were used in three separated BP recombination reactions between an att B-flanked DNA fragment and an att P-containing donor vector to generate an entry clone. We generated three entry clones: pENTR™L4-R1-Myo-3, pENTR™L1-L2-GFP and pENTR™L1-L2-*bmk-1*, and pENTR™R2-L3-3′-UTR.

**Step 3**: Expression clones were generated by LR reaction:

Expression clones were generated by LR reactions between an att L-containing entry clone and an att R-containing destination vector. Three entry clones from step 2 and the destination vector pDEST™R4-R3 were used together in a single LR reaction to generate the expression clones pCFJ150 with 3 fragments, which were named pCFJ150-GFP and pCFJ150-*bmk-1*.

#### Microinjection

A DNA mixture of 10 ng plasmid pCFJ150-GFP, and 10 ng plasmid pCFJ150-*bmk-1*, and 50 ng carrier plasmid pUC-19 was microinjected into the syncytial gonad of wild type N2 animals [17, 36]. After 48–72 hr, we scored the progeny of injected worms using a fluorescent stereomicroscope (Olympus S2 × 16). Each green transformed progeny was transferred to a separate NGM plate as an independent line by a worm pick. Only the lines for which the F1 progeny could pass the transgene to their progeny with efficiencies (green positive worms as a fraction of all progeny) greater than 50% were kept and used in further experiments. GFP alone with the carrier DNA were injected to obtain the control lines for these experiments. Each transgenic line was maintained by transferring 5–10 green worms to a new NGM plate every 3–4 days.

#### Genotyping

Single adult worms were picked and put into 10 ul lysis buffer (50 mM KCl, 10 mM Tris pH8.3, 2.5 mM MgCl2, 0.45% NP40, 0.45% Tween 20, 0.01% gelatin) with fresh 1.0 mg/ml Proteinase K. Worms were digested at 60°C for an hour and 95°C for 15 min. Digested lysate template was amplified by PCR using oligonucleotide primer sequences forward: 5′-ctatgaccatgattacgccaagc; and reverse: 5′-gatgatgaggattcacgacaca. The PCR product was indicated by a 3273bp band on a 1% agarose gel. The *bmk-1* over-expressing lines were all genotyped to ensure the over-epxressors were really over-expressing *bmk-1*.

#### Lifespan assay

To quantify lifespan, L4 larvae from the age-synchronized population of worms were transferred to NGM plates supplied with 100 ug/ml Ampicillin and 500 nM 5-fluoro-20-deoxyuridine (FUDR) seeded with sufficient OP50 bacteria. Worms were monitored by tapping their head with a platinum worm pick every 1 or 2 days until they were dead. Worms were scored as dead if they did not respond by moving the head to tapping. Worms which had fled or crawled off the agar and died on the side were censored and removed from analysis. At least three individual experiments were performed in each group.

#### Paraquat treatment and heat shock

Age-synchronized L4 larvae were first transferred to FUDR plates. After 24 hr, for the paraquat assay young adults were moved to FUDR plates supplied with 4 mM paraquat for the duration of the experiment [17]. For heat shock, the plates with young adult worms were moved to a 35°C incubator for 2 hr, and then removed back to 25°C conditions [17]. All of the worms were subsequently monitored every day for lifespan, and survival curves were based on daily counting.

#### UV radiation

Young adult worms were irradiated on NGM plates without OP50 under a germicidal bulb (254 nm) at 0.1 J/cm2 by using an UV crosslinkers. (CL-1000 Ultraviolet Crosslinkers, LLC Upland, CA, US). Then the worms were transferred to FUDR plates that were seeded with OP50. Worms were checked daily through their lives to generate survival curves [21].

#### RNAi induction

Gene knock down by RNAi was performed by feeding the worms with bacteria which produced dsRNA against the gene of interest. RNAi for *bmk-1* was a gift (Weidhaas lab). Briefly, on the first day, the RNAi clone in *E. coli* was incubated overnight at room temperature on RNAi agar plates with 25 μg/ml carbenicillin and 1 mM isopropylthiogalactosidase (IPTG) to induce dsRNA expression. On the second day, L4 larvae were transferred to the seeded plates to be monitored for their life spans. Bacteria containing RNAi empty vector were used as food for the control group [37].

### RNA isolation and quantitative PCR

Total RNA extraction was performed by using RNeasy mini kit from QIAGEN. 10 worms were collected as one sample in M9 buffer then were washed three times and excess M9 was carefully removed. The pellets were resuspended in 350 ul lysis buffer with β-mercaptoethanol, mix with equal volume of 70% ethanol. The mixture was transferred to a spin column and followed by the manufacturer protocol. DNase digestion was performed in the column and RNA was eluted in 13 ul RNase-free water. Reverse transcription was performed by using Ominscript kit (QIAGEN). For real-time PCR, each 25 ul reaction containing 12.5 μl of 2x SybrGreen supermix (Bio-Rad), 0.4 μM of each primer, and 2 μl of template cDNA was performed on a C1600 Thermal Cycler (Bio-Rad). Relative gene expression level was normalized to act-1 and calculated using the ΔΔCt (cycle threshold) method [39–40].

**Table:** 

	Cloning primers
attB1-bmk-1-F	5′-ggggacaagtttgtacaaaaaagcaggctcgttggattcgacaatggcatcc
attB2-bmk-1-R	5′-ggggaccactttgtacaagaaagctgggtctgtgcgttagttttcgaaatc
attB4-Pmyo3-F	5′-ggggacaactttgtatagaaaagttgaacggctataataagttctt
attB1r-Pmyo3-R	5′-ggggactgcttttttgtacaaacttgttctagatggatctagtgg
bmk-1-R	5′-tcaacttgaatgtggttctcc
Pmyo3-F	5′-caaatttctcggcgatttgt
	mRNA primers
bmk-1-F	5′-cgaaagttgcggagaatcat
bmk-1-R	5′-ttcacatcgcaagtctccac
hsp-16-F	5′-ggctcagatggaacgtcaa
hsp-16-R	5′-gcttgaactgcgagacattg
ced-3-F	5′-cggagttcctgcatttcttc
ced-3-R	5′-acagacggcttgaatgaacc
hsp-70-F	5′-tgaaaaggcacttcgtgatg
hsp-70-R	5′-ccaaaggctactgcttcgtc
hsp-12-F	5′-gtgatggctgacgaaggaac
hsp-12-R	5′-gggaggaagttatgggcttc
act-1-F	5′-tgctgatcgtatgcagaagg
act-1-R	5′-tagatcctccgatccagacg

#### Examination of the expression of Bmk-1 homologs in mouse and human tissues

Total RNA of mouse and human ovaries were isolated with the RNeasy mini kit (QIAGEN). Five young animals (3 months old) and 5 old animals (22 months old) were used for the gene expression analysis. Eight young humans (ages 18–25 yrs old) and 10 old humans (ages >60 yrs old) were included for this study. Gene expression was analyzed by Affymetrix gene array (version ST 1.0) and the differential expression of Bmk-1 homologs was judged by both fold change and by *p* value [17, 40].

## SUPPLEMENTARY TABLE AND FIGURES


