# Supplementary material for: Bmk-1 regulates lifespan in Caenorhabditis elegans by activating hsp-16
Source: Oncotarget. 2015 Jul 31;6(22):18790–9. doi: 10.18632/oncotarget.4618 (PMC4662456; doi:10.18632/oncotarget.4618)
Supplement: Supplementary file 1 [file oncotarget-06-18790-s001.pdf]

SUPPLEMENTARY TABLE AND FIGURES

Supplementary Table S1: Protein sequences of BMK-1/KIF11 across species

Gene Symbol	Gene Accession No.	Species
KIP1	P28742	*Saccharomyces cerevisiae*
Bmk-1	NP_001256587.1	*Caenorhabditis elegans*
Klp61F	NP_476818.1	*Drosophila melanogaster*
Kif11	NP_775368.1	*Danio rerio*
Kif11	NP_034745.1	*Mus musculus*
Kif11	NP_001162583.1	*Rattus norvegicus*
KIF11	XP_534964.3	*Canis lupus familiaris*
AT2G37420	NP_850281.1	*Arabidopsis thaliana*
Kif11	NP_001016116.2	*Arabidopsis tropicalis*
KIF11	XP_507923.2	*Arabidopsis troglodytes*
KIF11	NP_004514.2	*Homo sapiens*

Note: Gene Symbols and Gene Accessible Numbers are from RefSeq database of National Center for Biotechnology Information, NIH (http://www.ncbi.nlm.nih.gov/refseq/).

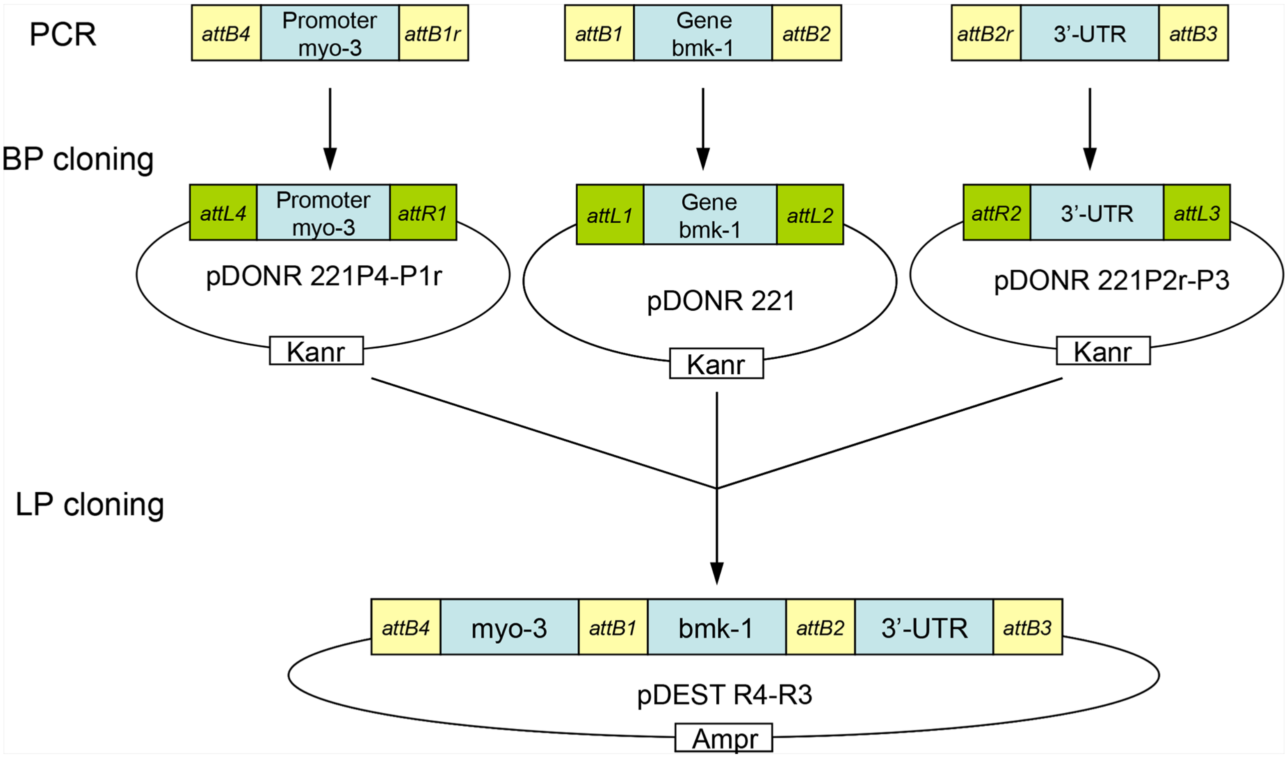

Supplementary Figure S1: Construction of *bmk-1* over-expression plasmid map.

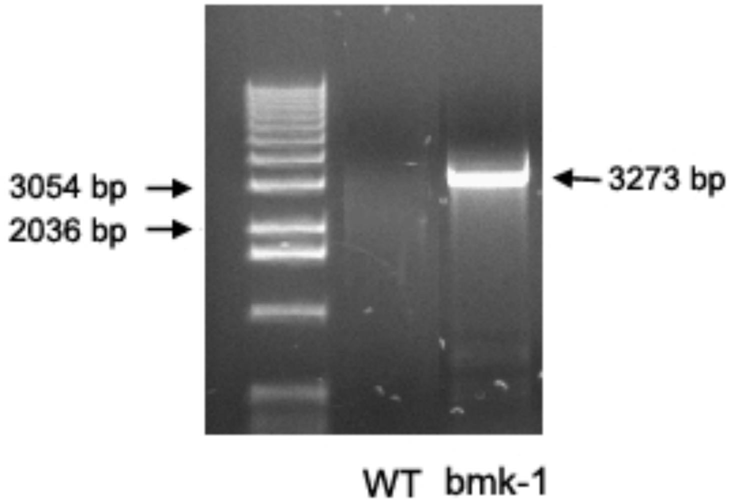

Supplementary Figure S2: Genotyping of *bmk-1* over-expression worm lines.

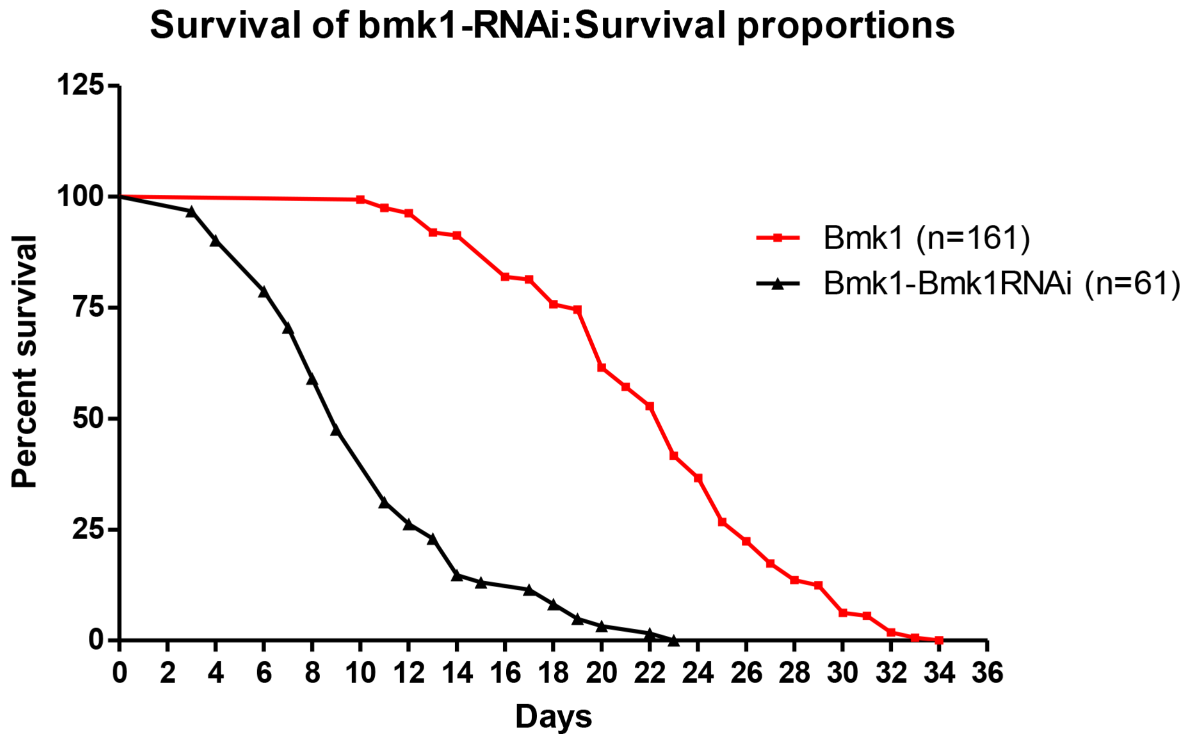

Supplementary Figure S3: Survival of bmk-1 RNAi on bmk-1 over-expression worm lines.
